# Subthreshold Micropulse Laser at Two Wavelengths for Juvenile X-Linked Retinoschisis: A Two-Patient Feasibility Study with 8-Month Follow-Up

**DOI:** 10.3390/jcm15187093

**Published:** 2026-09-13

**Authors:** Pietro Emanuele Napoli, Matteo Nioi

**Affiliations:** 1 Studio Oculistico Dr. Pietro Emanuele Napoli, 09014 Carloforte, Sardinia, Italy; pietronapoli@ymail.com; 2 Forensic Medicine Unit, Department of Medical Sciences and Public Health, University of Cagliari, 09040 Cagliari, Sardinia, Italy

**Keywords:** X-linked retinoschisis, *RS1*, subthreshold micropulse laser, maculopathy, optical coherence tomography, retinal dystrophy

## Abstract

**Background:** Juvenile X-linked retinoschisis (XLRS; OMIM 300839) is an inherited retinal disorder characterized by foveal schisis and variable visual impairment. There is no approved disease-modifying treatment, and evidence for interventions targeting XLRS-associated macular changes remains limited. **Objective:** To assess the feasibility, short-term safety, and descriptive anatomical and functional outcomes of subthreshold micropulse laser (SMPL) delivered at two wavelengths in two brothers with molecularly confirmed XLRS. **Methods:** Two brothers aged 31 and 33 years, both hemizygous for the *RS1* variant c.589C>T (p.Arg197Cys), underwent bilateral SMPL in a single session. Each right eye received 520-nm green SMPL (300 mW), and each left eye received 577-nm yellow SMPL (350 mW). The remaining parameters were matched, as follows: 100 µm spot diameter, 200-ms exposure, 5% duty cycle, zero spot spacing, and 400 confluent applications across the macula. Best-corrected visual acuity (BCVA) and central foveal thickness (CFT) were assessed at baseline and at months 4 and 8. **Results:** Descriptively, individual-eye measurements showed heterogeneous anatomical and visual changes over follow-up. In patient 1, right-eye CFT changed from 485 to 466 and 153 µm, while BCVA changed from 3/10 to 4/10 and 6/10 at months 4 and 8, respectively. Left-eye CFT changed from 281 to 209 and 209 µm, while BCVA changed from 2/10 to 3/10 and 4/10. In patient 2, right-eye CFT changed from 488 to 250 and 453 µm, with BCVA changing from 2/10 to 4/10 and 4/10. Left-eye CFT changed from 398 to 249 and 498 µm, with BCVA changing from 2/10 to 3/10 and 3/10. No treatment-related adverse events or visible changes on infrared reflectance or fundus autofluorescence imaging were observed through month 8. **Conclusions:** A single bilateral SMPL session was feasible and was followed by transient or sustained anatomical and visual changes in these two patients. Because this uncontrolled case series included only two related participants and assigned wavelength by eye laterality with different power settings, it cannot establish efficacy or comparative wavelength superiority. The findings are hypothesis-generating and warrant evaluation in a prospective controlled study.

## 1. Introduction

Juvenile X-linked retinoschisis (XLRS; OMIM 300839) is an early-onset inherited retinal disorder characterized by splitting of the neurosensory retina, typically at the fovea and sometimes in the peripheral retina. Haas first described the condition in 1898, and Jager introduced the term “retinoschisis” in 1953. The terms X-linked congenital retinoschisis and juvenile X-linked retinoschisis are commonly used for the same disorder [1]. XLRS is usually inherited as an X-linked recessive trait and therefore predominantly affects males, although symptomatic females have been reported [2,3]. Its estimated prevalence ranges from approximately 1 in 5000 to 1 in 25,000 individuals [1]. The causative gene, RS1, is located at Xp22 and encodes retinoschisin, a 224-amino-acid extracellular protein containing a discoidin domain. Retinoschisin is secreted mainly by photoreceptors and bipolar cells and contributes to retinal cellular adhesion and structural organization [4,5,6]. The mechanisms that produce schisis cavities remain incompletely understood.

The classic macular phenotype consists of a spoke-wheel appearance produced by intraretinal schisis cavities, most commonly involving the inner nuclear layer but potentially extending across several retinal layers. Peripheral schisis, vitreous hemorrhage, retinal detachment, and progressive macular atrophy contribute to the broad phenotypic spectrum. Full-field electroretinography may show a reduced b-wave relative to the a-wave, although this finding is neither universal nor sufficient for diagnosis. Accordingly, contemporary diagnosis integrates clinical examination, multimodal retinal imaging, electrophysiology when indicated, and molecular confirmation [1]. The clinical course is heterogeneous even among relatives carrying the same variant. Visual acuity may remain relatively stable for years, whereas structural changes can fluctuate or progress independently of measured acuity. Optical coherence tomography (OCT) is particularly valuable because it can reveal retinal splitting when funduscopic findings are subtle, localize the involved retinal layers, quantify foveal thickness, and document changes over time [7]. This variability also complicates the interpretation of uncontrolled treatment observations, as follows: a post-treatment anatomical change may reflect an intervention, spontaneous fluctuation, measurement variability, or a combination of these factors.

No approved disease-modifying treatment for XLRS is currently available. Carbonic anhydrase inhibitors (CAIs) are used off label in selected patients and may reduce cystoid fluid collections, although functional benefit is variable and often modest [8,9,10]. Gene-augmentation strategies remain investigational; a recent early clinical study of subretinal RS1 gene therapy reported an acceptable short-term safety profile and supported further clinical evaluation [11].

Subthreshold micropulse laser (SMPL) delivers energy in short pulse trains separated by cooling intervals. Unlike conventional continuous-wave photocoagulation, SMPL is intended to produce a biological response without a clinically visible retinal burn or scar [12,13]. Proposed mechanisms include modulation of heat-shock proteins, inflammatory mediators, and retinal pigment epithelium (RPE) and Müller-cell homeostasis [14,15,16]. These effects have principally been investigated in acquired macular diseases, and their specific role in XLRS has not yet been established. Nevertheless, the absence of an intentionally destructive endpoint and the possibility of influencing retinal fluid homeostasis provide a rationale for cautious feasibility testing.

Green and yellow SMPL systems are used for several macular disorders, but the biological response depends on wavelength, tissue absorption, power, exposure duration, duty cycle, spot size, treatment density, and titration strategy [17]. Nominal power alone therefore does not establish equivalent retinal exposure across devices. Any comparison between wavelengths must control these parameters and account for baseline differences between eyes.

This report describes the anatomical, functional, and short-term safety course after bilateral SMPL in two brothers with XLRS caused by the RS1 c.589C>T (p.Arg197Cys) variant. The right and left eyes were treated with 520-nm and 577-nm SMPL, respectively. The objectives were to document procedural feasibility, report eye-level outcomes at 4 and 8 months, and identify methodological considerations for a future controlled study. The treatment allocation was not randomized, and the observations were intended to be descriptive rather than a formal test of efficacy or wavelength superiority.

## 2. Materials and Methods

### 2.1. Study Design and Ethical Approval

This exploratory two-patient clinical case series was conducted at the Eye Clinic, Department of Surgical Sciences, University of Cagliari, Italy. The two participants included in this report had been enrolled in a broader prospective clinical research program approved by the competent institutional ethics committee in July 2014. They constituted a subset of the population enrolled in that approved program. Before enrollment, both participants received written information describing the study aims and procedures and signed informed-consent forms covering study participation, the collection and processing of clinical data, and the anonymous publication of clinical findings and retinal images. All procedures were conducted in accordance with the Declaration of Helsinki and Good Clinical Practice principles.

### 2.2. Participants and Diagnostic Characterization

An Italian four-generation family comprising 11 examined individuals had previously undergone clinical, ophthalmological, electrophysiological, and molecular assessment for an undetermined inherited retinal dystrophy [8]. Two affected brothers, aged 31 years (patient 1) and 33 years (patient 2), were included in the present report. Sequence analysis, performed as previously described [8], showed that both were hemizygous for the c.589C>T (p.Arg197Cys) missense variant in exon 6 of *RS1*. During the 24 months preceding SMPL treatment, neither patient had received treatment for XLRS, including carbonic anhydrase inhibitors, and serial clinical and OCT assessments had substantial macular structural fluctuations. At baseline, both patients underwent medical-history review and comprehensive bilateral ophthalmic assessment. The examination included best-corrected visual acuity (BCVA), slit-lamp biomicroscopy, dilated fundus examination, near-infrared reflectance imaging, fundus autofluorescence (FAF), fluorescein angiography, and spectral-domain OCT (SD-OCT) using the SPECTRALIS HRA + OCT platform (Heidelberg Engineering GmbH, Heidelberg, Germany). XLRS was diagnosed from the combined findings of reduced BCVA, a characteristic stellate macular appearance, intraretinal schisis cavities on OCT, the family history, and the molecularly confirmed RS1 variant. The same clinical definitions were used for both patients.

### 2.3. Treatment Allocation and Laser Procedure

Both patients underwent bilateral SMPL in a single session performed by the same surgeon. Treatment assignment was fixed by laterality, as follows: the right eye received the 520-nm protocol and the left eye received the 577-nm protocol. This allocation was not randomized and was not designed as an independent comparative trial. It provided paired descriptive observations while preserving the same treatment assignment in both participants. Before treatment, the pupils were dilated with tropicamide 0.5% and phenylephrine hydrochloride 10% eye drops. Topical oxybuprocaine hydrochloride 0.4% was used for corneal anesthesia. A Mainster macular contact lens (Ocular Instruments, Inc., Bellevue, WA, USA) was placed on the cornea with the aid of viscoelastic. The right eye was treated with a 520-nm green SMPL (LEAF, Norlase ApS, Ballerup, Denmark), whereas the left eye was treated with a 577-nm yellow SMPL (Easyret, Quantel Medical, Cournon-d’Auvergne, France).

Power was selected using a burn-titration approach based on previously described SMPL methodology [18]. Titration was performed separately for each laser device in micropulse mode in a healthy retinal area away from the treatment target. Power was increased to identify the minimum threshold producing a barely visible retinal burn and was subsequently reduced below this visible threshold for treatment. Using this procedure, the selected treatment powers were 300 mW for the 520-nm system and 350 mW for the 577-nm system. No visible retinal endpoint was sought during treatment.

The right eye of each patient was treated with a 520-nm green SMPL system (LEAF, Norlase, Ballerup, Denmark) using 300 mW, and the left eye was treated with a 577-nm yellow SMPL system (Easyret, Quantel Medical, Cournon-d’Auvergne, France) using 350 mW. For both systems, spot diameter was 100 μm, envelope exposure time was 200 ms, duty cycle was 5%, and spot spacing was zero. Under minimal slit-lamp illumination, the macular treatment area was covered with 400 confluent applications. Spot size, exposure time, duty cycle, spacing, and number of applications were therefore matched, whereas wavelength, device, and nominal power differed (Table 1).

For reproducibility, the calculated nominal active-emission energy per application was 3.0 mJ for the 520-nm protocol and 3.5 mJ for the 577-nm protocol, using displayed power × envelope duration × duty cycle. These calculated values describe the programmed exposure and should not be interpreted as direct measurements of retinal energy absorption.

### 2.4. Outcome Definitions and Follow-Up

Feasibility was evaluated from completion of the planned bilateral treatment and scheduled follow-up. Safety assessment comprised clinical examination and review of near-infrared reflectance and FAF images for visible treatment-related changes, together with documentation of reported or observed adverse events. Anatomical response was described using central foveal thickness (CFT), and functional response was described using BCVA.

**Figure 1:**
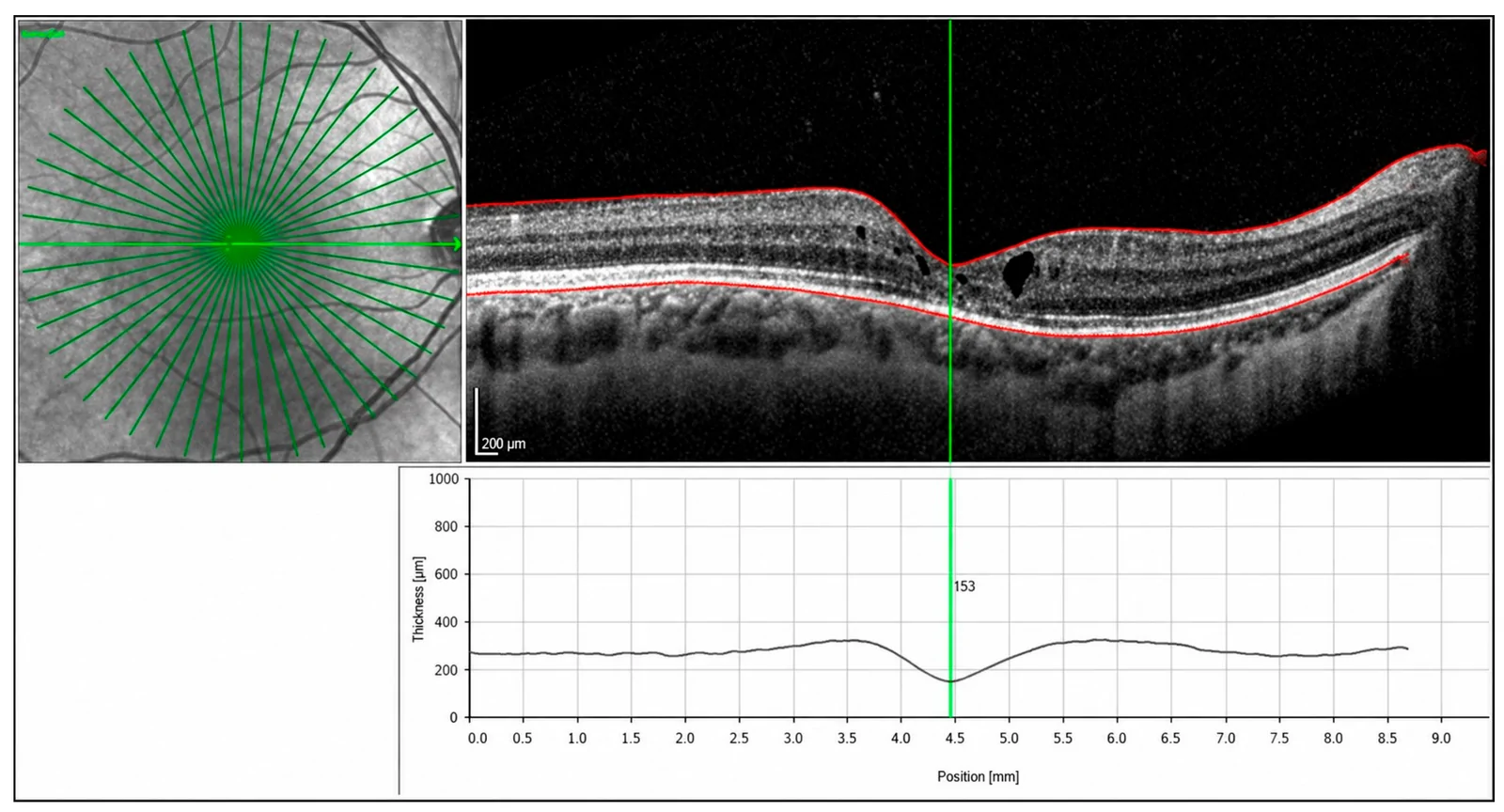
Quantitative SD-OCT assessment. (**Top left**): radial scan pattern used to localize the foveal intersection. (**Top right**): corresponding B-scan. (**Bottom**): retinal-thickness profile used to determine central foveal thickness.

BCVA, near-infrared reflectance, FAF, and SD-OCT were reassessed at months 4 and 8. All longitudinal SD-OCT examinations were performed using the same SPECTRALIS HRA + OCT platform and acquisition protocols. The device’s integrated eye-tracking and follow-up functions were used to reproduce the same retinal location across examinations. CFT was automatically calculated using the integrated SPECTRALIS software (HEYEX, version 1.9.201.0; Heidelberg Engineering GmbH, Heidelberg, Germany) as retinal thickness at the foveal intersection of 24 radial scans (Figure 1).

Decimal BCVA values were converted to logarithm of the minimum angle of resolution (logMAR) using −log10(decimal acuity) and were reported in both formats. Percentage CFT change was calculated relative to baseline as [(follow-up CFT–baseline CFT)/baseline CFT] × 100. Given the sample size and relatedness of the participants, no inferential statistical analysis, pooling across eyes, or imputation was performed. Each eye was reported longitudinally, and percentage changes were used only to aid descriptive interpretation rather than to imply statistical significance.

## 3. Results

Both patients completed the planned bilateral treatment and the month-4 and month-8 assessments, providing complete BCVA and CFT observations for all four eyes. The procedure was therefore feasible as delivered. During the preceding 24 months, serial clinical and OCT assessments had not shown substantial macular structural fluctuations in either patient. All eyes showed some reduction in CFT at month 4, but the magnitude and durability of the anatomical response differed between patients and eyes. The following findings are reported descriptively at the individual-eye level.

### 3.1. Patient 1

**Figure 2:**
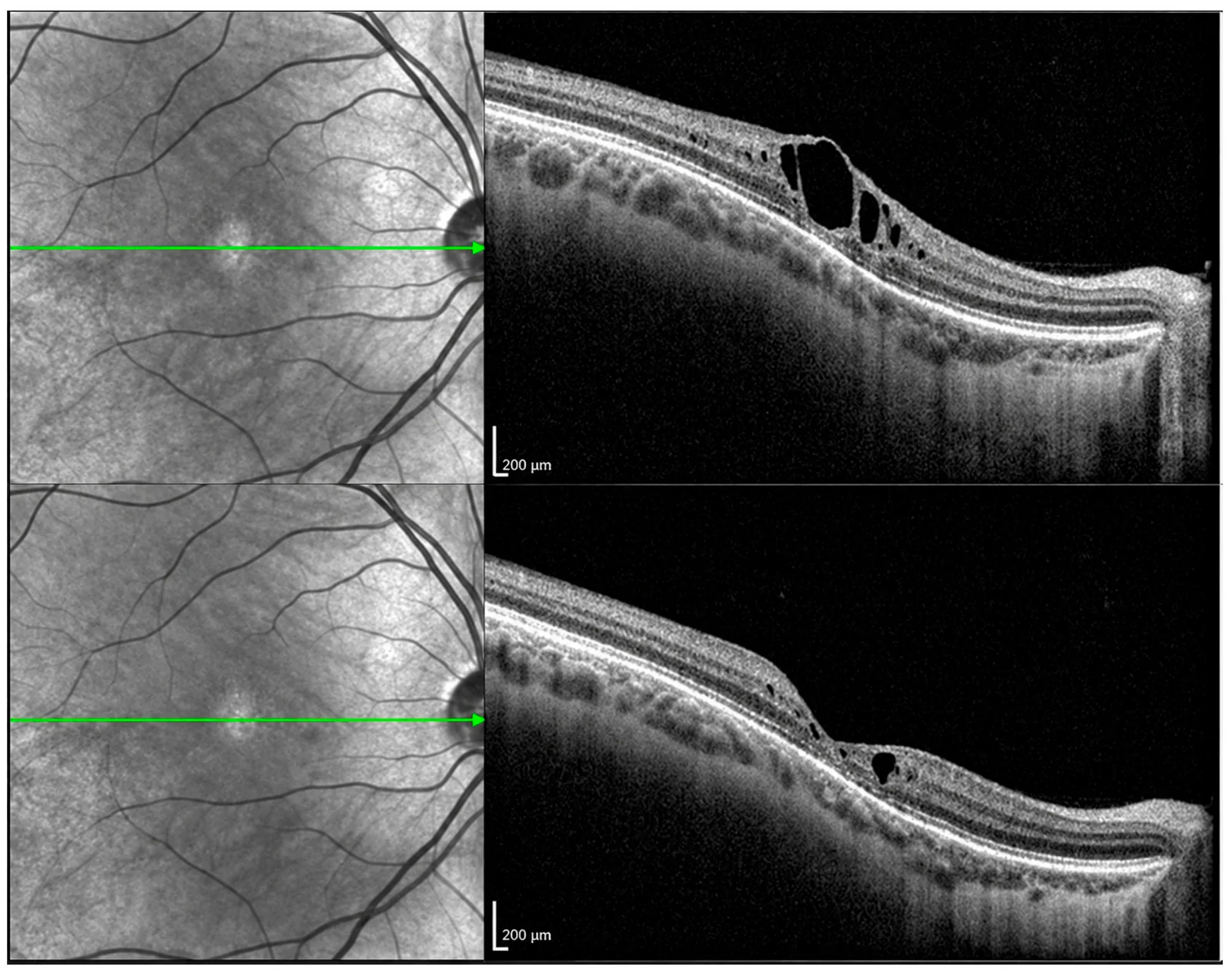
Representative SD-OCT images of the right eye of patient 1. (**Top**): baseline scan, with BCVA of 3/10 and CFT of 485 µm. (**Bottom**): month 8 scan, showing reduced schisis and CFT of 153 µm, with BCVA of 6/10.

In patient 1, baseline CFT was 485 μm in the right eye and 281 μm in the left eye; BCVA was 3/10 (0.522 logMAR) and 2/10 (0.698 logMAR), respectively. At month 4, CFT was 466 μm in the right eye (−3.9%) and 209 μm in the left eye (−25.6%), while BCVA improved to 4/10 (0.397 logMAR) and 3/10 (0.522 logMAR). At month 8, right-eye CFT had decreased to 153 μm (−68.4%), with BCVA of 6/10 (0.221 logMAR); left-eye CFT remained 209 μm (−25.6%), with BCVA of 4/10 (0.397 logMAR). Representative right-eye SD-OCT images are shown in Figure 2.

Between months 4 and 8, the right eye showed a further 313 μm CFT reduction and a two-line decimal-acuity increase from 4/10 to 6/10. The left-eye CFT remained stable over the same interval, while decimal BCVA increased from 3/10 to 4/10. Thus, the largest anatomical change in this patient occurred after the first scheduled post-treatment assessment rather than during the initial 4 months.

### 3.2. Patient 2

In patient 2, baseline CFT was 488 μm in the right eye and 398 μm in the left eye, with BCVA of 2/10 (0.698 logMAR) in both eyes. At month 4, CFT decreased to 250 μm in the right eye (−48.8%) and 249 μm in the left eye (−37.4%); BCVA improved to 4/10 (0.397 logMAR) and 3/10 (0.522 logMAR), respectively. At month 8, CFT had increased to 453 μm in the right eye (−7.2% relative to baseline) and 498 μm in the left eye (+25.1%), while BCVA remained at the month-4 values. From month 4 to month 8, CFT increased by 203 μm in the right eye and 249 μm in the left eye. The right eye nevertheless remained slightly thinner than at baseline, whereas the left eye became thicker than at baseline. BCVA did not decline with this anatomical recurrence, illustrating that structural and functional changes were not strictly parallel over the available follow-up.

### 3.3. Safety Observations

No visible treatment-related changes were detected on near-infrared reflectance or FAF imaging at either follow-up visit. No treatment-related adverse events were reported or observed through month 8. Because only four eyes were exposed, these findings describe the observed short-term course and do not establish the frequency of uncommon adverse effects (Table 2 and Table 3).

## 4. Discussion

This case series describes the clinical course of four eyes in two brothers after a single bilateral SMPL session. Treatment was technically feasible, and no treatment-related adverse events were observed during 8 months of follow-up. Both patients showed anatomical and visual changes after treatment, but their time courses differed. Patient 1 showed continued improvement through month 8, most prominently in the green-treated right eye. Patient 2 showed the largest CFT reduction at month 4, followed by partial or complete anatomical recurrence by month 8, while BCVA remained at the month-4 level. In particular, the left-eye CFT of patient 2 increased from 398 μm at baseline to 498 μm at month 8, emphasizing the heterogeneous and potentially transient nature of the observed anatomical changes.

To our knowledge, this is the first report specifically focused on SMPL for schitic macular changes attributed to XLRS and using two wavelengths in paired eyes. A previous case report described successful SMPL treatment of coexisting bilateral central serous chorioretinopathy in a patient with XLRS; that intervention targeted the accompanying chorioretinopathy rather than XLRS-associated schisis itself [19]. The present findings therefore extend, but do not establish, the potential role of SMPL in this clinical setting. XLRS results from dysfunction of retinoschisin, an extracellular protein important for retinal organization and intercellular adhesion. SMPL cannot correct the underlying genetic defect. Any effect would instead be indirect. Experimental and clinical studies in other retinal disorders suggest that subthreshold stimulation may alter heat-shock-protein expression, inflammatory signaling, and fluid handling by the RPE and Müller cells [14,15,16]. These mechanisms may influence intraretinal fluid and schisis-cavity dimensions, although their specific role in XLRS has not yet been established. CAIs are currently the most frequently described pharmacological approach to XLRS-associated cystoid or schitic macular changes. Their anatomical effects are variable, and visual improvement may be limited. In a recent 42-patient retrospective cohort, CAI treatment was associated with reduced CFT, whereas the average visual-acuity change was modest and not clinically meaningful; 17% of patients discontinued treatment because of adverse effects [10]. Neither patient in the present study received CAI therapy or other treatment for XLRS during the study period or during the preceding 24 months. SMPL might eventually be studied as a local, nonpharmacological option for selected patients, but the present observations are insufficient to support clinical adoption or a retreatment schedule. The anatomical recurrence observed in patient 2 raises the hypothesis that any SMPL-associated anatomical effect, if confirmed, may be transient after a single treatment session. Whether repeated treatment could produce a more durable or cumulative response cannot be determined from the present observations and should be evaluated in future prospective studies.

The right eyes in this report showed numerically larger maximum CFT reductions than the left eyes. This pattern must not be interpreted as evidence that 520-nm treatment is superior to 577-nm treatment. Wavelength was assigned deterministically by laterality; only two patients were treated, baseline CFT differed between fellow eyes, and power differed between devices (300 versus 350 mW). Although the calculated nominal active-emission energy differed between protocols (3.0 versus 3.5 mJ per application), the treatment powers had been determined independently for each device by titration to the same predefined visible threshold. These nominal calculated values therefore should not be interpreted as matched—or directly comparable—retinal biological exposure. Consequently, wavelength, eye laterality, baseline anatomy, device, power, and nominal energy remain inseparable in the present design. A valid wavelength comparison would require randomization, standardized dosimetry, a larger sample, and masked outcome assessment. Several additional limitations should be emphasized. First, the participants were two related adults carrying the same *RS1* variant, which limits generalizability across ages, genotypes, and phenotypes. Second, the absence of an untreated or sham-treated control prevents separation of treatment-associated change from spontaneous fluctuation, measurement variability, or regression to the mean. Although serial clinical and OCT assessments during the preceding 24 months had not shown substantial macular structural fluctuations in either patient, this historical stability cannot exclude spontaneous fluctuation after treatment and cannot substitute for a concurrent control group. Third, outcomes were recorded at only three time points over 8 months, and no prespecified retreatment protocol was used. Fourth, decimal visual acuity rather than standardized ETDRS letter scores was reported, and post-treatment electrophysiology, microperimetry, and patient-reported outcomes were unavailable. Although both patients reported subjective visual benefit, no standardized patient-reported outcome instrument was administered. Finally, the very small sample can identify neither uncommon adverse events nor reliable predictors of response. Future studies should use a prospective protocol with randomized eye-level wavelength assignment or a parallel control group, standardized power titration, masked OCT grading, repeated CFT or schisis-volume measurements, ETDRS BCVA, functional testing, including microperimetry and electrophysiology, standardized patient-reported outcome measures, and longer follow-up. Stratification by age, baseline retinal structure, and *RS1* genotype would help determine whether any response is reproducible and clinically meaningful. Prospective studies should also determine whether repeated SMPL sessions provide any reproducible cumulative or sustained anatomical effect.

## 5. Conclusions

In two brothers with the *RS1* c.589C>T (p.Arg197Cys) variant, a single bilateral SMPL session was feasible and was followed by transient or sustained anatomical and visual changes without observed treatment-related adverse events through month 8. The response was heterogeneous and, in one patient, anatomically transient. These uncontrolled observations do not demonstrate efficacy and do not permit a comparison of green and yellow wavelengths. They provide a rationale for a carefully controlled prospective study rather than a basis for routine clinical treatment.

## Figures and Tables

**Table 1:** Subthreshold micropulse laser treatment parameters. RE, right eye; LE, left eye.

Parameter	RE Protocol	LE Protocol
Wavelength and device	520 nm; LEAF (Norlase)	577 nm; Easyret (Quantel Medical)
Displayed power	300 mW	350 mW
Spot diameter	100 μm	100 μm
Envelope exposure	200 ms	200 ms
Duty cycle	5%	5%
Calculated active-emission energy/application	3.0 mJ	3.5 mJ
Spot spacing	0 (confluent)	0 (confluent)
Applications	400	400
Treatment area	Macula	Macula

**Table 2:** Best-corrected visual acuity at baseline and after treatment. Values are reported as decimal acuity with logMAR in parentheses. RE, right eye; LE, left eye.

Patient	Eye (Wavelength)	Baseline	Month 4	Month 8
1	RE (520 nm)	3/10 (0.522)	4/10 (0.397)	6/10 (0.221)
1	LE (577 nm)	2/10 (0.698)	3/10 (0.522)	4/10 (0.397)
2	RE (520 nm)	2/10 (0.698)	4/10 (0.397)	4/10 (0.397)
2	LE (577 nm)	2/10 (0.698)	3/10 (0.522)	3/10 (0.522)

**Table 3:** Central foveal thickness at baseline and after treatment. Follow-up values include percentage change from baseline in parentheses. CFT, central foveal thickness; RE, right eye; LE, left eye.

Patient	Eye (Wavelength)	Baseline CFT	Month-4 CFT	Month-8 CFT
1	RE (520 nm)	485 μm	466 μm (−3.9%)	153 μm (−68.4%)
1	LE (577 nm)	281 μm	209 μm (−25.6%)	209 μm (−25.6%)
2	RE (520 nm)	488 μm	250 μm (−48.8%)	453 μm (−7.2%)
2	LE (577 nm)	398 μm	249 μm (−37.4%)	498 μm (+25.1%)

## Data Availability

The data presented in this study are available on reasonable request from the corresponding author. The data are not publicly available because of privacy and ethical restrictions related to clinical and genetic information concerning members of the same family.
